# Computational Methods for the Analysis of Genomic Data and Biological Processes

**DOI:** 10.3390/genes11101230

**Published:** 2020-10-20

**Authors:** Francisco Gómez-Vela, Federico Divina, Miguel García-Torres

**Affiliations:** Computer Science Division, Pablo de Olavide University, ES-41013 Seville, Spain; fdivina@upo.es (F.D.); mgarciat@upo.es (M.G.-T.)

Today, new technologies, such as microarrays or high-performance sequencing, are producing more and more genomic data. This fact has brought new opportunities and challenges in the fields of computational biology and bioinformatics, since this huge number of data need to be analysed in order to be exploited.

In this context, new computational methods and tools, such as machine learning approaches or gene expression analysis tools, could provide the solution to such issues.

The overall aim of this Special Issue is to compile the latest research and developments in the field of computational methods for the analysis of gene expression data and, in particular, with the modelling of biological processes.

Among all the submissions, eleven papers were accepted and published in this Special Issue. In this sense, machine learning-based approaches have received particular attention, such as the work presented by Vanhaeren et al. [1]. In this work, the authors used 1D sequencing signals to model cohesin-mediated chromatin interactions in two human cell lines and evaluate the prediction models obtained. To this end, they tested the performance of six popular machine learning algorithms: decision trees, random forests, gradient boosting, support vector machines, multi-layer perceptron and deep learning. The results obtained showed that gradient boost outperformed the other five methods, yielding accuracies of about 95%. Despite these results, the authors established that it was necessary to examine other cell lines and tissues to confirm the obtained observations.

In another article, Rehman and Chong [2] presented work where the authors propose a Convolution Neural Network (CNN) and Long Short-Term Memory (LSTM)-based tool named DNA6mA-MINT for DNA-6mA modification identification. The tool uses the CNN for feature extraction, while LSTM provides optimal interpretation for those features. The authors showed that the performance of their tool is superior to that achieved with existing state-of-the-art techniques on the “combined-species”, *Mus musculus* genome, and rice genome datasets. Moreover, the authors carried out a performance analysis on 5- and 10-fold cross-validation in order to obtain a better comparative analysis. The tool is provided by a user-friendly web server, publicly available.

Another example of the use of CNN is the work by Mahmoudi et al. [3], where a new computational model for identifying N6-methyladenosine (m6A) post-transcriptional modification in RNAs is proposed. The technique, called iMethyl-deep, provides a novel computational method for identifying m6A *Saccharomyces cerevisiae* sites by using single-nucleotide resolution to convert RNA sequences into high-quality feature representations in the CNN. The model is able to extract the relevant features from the input samples. The results obtained show that iMethyl-deep outperforms state-of-the-art methods and achieves accuracies of 89.19% and 87.44% on the M6A2614 and M6A6540 benchmark datasets, respectively.

In the context of machine learning, Delgado-Chaves et al. [4] presented an analysis of the effects of the gene *Ly6E* in the immune response against the coronavirus responsible for murine hepatitis (MHV). With this aim, the work used different datasets from mice, with and without the ablation of the gene *Ly6E*, to reconstruct computational gene co-expression networks, by using a machine learning-based algorithm called EnGNet. The authors carried out an integration of differential expression analyses and reconstructed network exploration, and significant differences in the immune response to the virus were observed in Ly6E compared to in wild-type animals. The obtained results show that Ly6E ablation in hematopoietic stem cells (HSCs) leads to a progressive impaired immune response in both the liver and spleen.

Zahoor and Zafar [5] propose a novel optimization algorithm inspired by the “infiltration tactics” of the war zone (ITO) for the task of classifying microarray datasets. The algorithm integrates parameter-free and parameter-based classifiers to provide a highly accurate and reliable binary classifier. The results are generated in two steps: (a) the Lightweight Infantry Group converges quickly to find non-local maxima and produces comparable results, and (b) the Follow-up Team (FT) applies advanced tuning to enhance the baseline performance. Each soldier is considered as a base model with its own independently chosen subset selection method (pre-processing, and validation methods and classifier). Therefore, successful soldiers are combined for optimal results. The performance of the algorithm was successfully tested using three mouse livers and a rat liver.

In this Special Issue, some tools are presented as well, such as in the work by Zeng et al. [6]. In this article, the authors present a new meta-analytic integration tool, called the Comparative Pathway Integrator (CPI), which is able to deal with multiples studies of different conditions. To do so, the tool uses an adaptive weighted Fisher’s method to discover consensual and differential enrichment patterns, a tight clustering algorithm to reduce pathway redundancy, and a text-mining algorithm to assist the interpretation of the pathway clusters. The authors demonstrate its effectiveness by applying CPI to jointly analyse six psychiatric disorder transcriptomic studies. The results described show functions confirmed by previous biological studies as well as novel enrichment patterns. The tool is publicly available as a CPI R package.

Another interesting tool provided as an R package, named metaRE, was presented by Novikova et al. [7]. MetaRE is able to perform a systematic search for cis-regulatory elements enriched in the promoters of the genes significantly changed in their transcription in a reaction. metaRE extracts datasets of multiple expression profiles generated to test the response of the same organism and identifies simple and composite cis-regulatory elements that are systematically associated with the differential expression of genes. The authors tested metaRE’s performance for the identification of low-temperature-responsive cis-regulatory code in *Arabidopsis thaliana* and *Danio rerio*. MetaRE was able to identify potential binding sites for known as well as unknown cold response regulators.

Computationally based analyses of biological processes are also included in this Special Issue. For example, the work by Agioutantis et al. [8] analysed 21 human hepatocellular carcinoma (HCC) cell lines (HCC lines) to explore intertumoral molecular diversity and pertinent drug sensitivity. This article proposes an integrative computational approach based on an exploratory and single-sample gene-set enrichment analysis of transcriptome and proteome data, and then a correlation analysis of drug-screening data. The presented results classified HCC lines into two groups. In particular, the lines were classified as poorly differentiated and well-differentiated, displaying lower/higher enrichment scores in a “Specifically Upregulated in Liver” gene set, respectively. It is worth mentioning that the analysis of correlation showed a differential effectiveness of specific drugs against poorly differentiated compared to well-differentiated HCC lines, which is possibly applicable in clinical research with patients with analogous features. As a result, this study may expand the knowledge of HCC lines and proposes a cost-effective computational approach to precision anti-HCC therapies. 

The work by Tian et al. [9] addresses the effect that deleting single-nucleotide polymorphisms (SNPs) of genes affected by large-effect expression Quantitative Trait Loci (eQTL) may have on gene expression. To this end, CRISPR-Cas9 mutagenesis was used to delete SNPs, obtaining single-cell clones. The bottlenecks for the fine mapping of such SNPs were suggested to be the impossibility of targeting many SNPs and the clonal variability of single-cell clones, among others. 

An analysis of the hypoxia inducible domain family member 2A (HIGD2A) was presented in Salazar et al. [10]. The protein HIG2A is produced by the HIGD2A gene, found in mitochondria and the nucleus, promoting cell survival in hypoxic conditions. The main objective of this study was to carry out a biosystem analysis of *HIGD2A* with the aim of discovering its implications in cancer biology. The authors used different public databases such Gene Expression Omnibus to evaluate some gene expression datasets. The results presented suggested that the gene’s alterations are present in the different cancers studied.

Finally, Ghanemi et al. [11] presented a review about the therapeutic alternatives to exercise in obesity. The review focuses on a functional genomics perspective, in particular, finding potential therapeutic targets for obesity. The authors point out various approaches, identifying differential gene expression-based studies that aimed at finding genes that are differentially expressed under diverse conditions depending on physical activity and diet (mainly high-fat). The authors suggested that this area of functional genomics-related exploration will lead to novel mechanisms and also new applications and implications along with a new generation of treatments for obesity and the related metabolic disorders.
